# Impact of routine pre‐operative risk assessment on patients undergoing emergency major abdominal surgery in a regional Victorian hospital

**DOI:** 10.1111/ans.19260

**Published:** 2024-10-28

**Authors:** Jason Douglas Cox, Frank Dunley, Jia Tian, Kate Booth, Jessica Paynter, Chun Hin Angus Lee

**Affiliations:** ^1^ Bendigo Health Monash University School of Rural Health Bendigo Victoria Australia; ^2^ Bendigo Health Monash University Bendigo Victoria Australia; ^3^ Bendigo Health Bendigo Victoria Australia

**Keywords:** abdominal surgery, emergency surgery, general surgery, quality improvement

## Abstract

**Background:**

Routine preoperative risk assessment (RPRA) using objective risk prediction tools may improve the perioperative outcomes of emergency major abdominal surgery (EMAS). This project aims to identify whether the introduction of RPRA with the ‘National Emergency Laparotomy Audit (NELA) Calculator’ as standard‐of‐care for EMAS at a regional Victorian hospital has improved postoperative outcomes, reduced unplanned postoperative critical care unit (CCU) admission rates, and impacted the ‘no‐lap’ rate.

**Methods:**

An audit was performed including all adult general surgery patients who required EMAS at Bendigo Health between September 2017 and August 2022, including those palliated up‐front. Patients requiring surgery for uncomplicated appendicitis, cholecystitis, trauma, and diagnostic laparoscopy were excluded. Patient demographics, preoperative NELA score, CCU admission data and postoperative outcomes were collected and compared between patients undergoing surgery before and after the introduction of RPRA.

**Results:**

Six hundred and ninety‐one patients were included in the analysis. Median NELA score was 5 (IQR 1.5–11.75). 2.60% of patients were palliated up‐front and did not proceed to surgery. Among the 673 operative patients, 30‐day mortality was 5.20%. Following the introduction of RPRA there was a significant reduction in the unplanned CCU admission rate, from 9.14% to 3.48% (*P* = 0.04). There was no change in postoperative mortality, severe complication rate or planned CCU admission rate.

**Conclusion:**

RPRA reduced rate of unplanned CCU admissions. Postoperative mortality and complication rates did not change following introduction of RPRA. RPRA appears useful in guidance of preoperative palliative decision‐making, but further study is required to validate its use in this context.

## Graphical abstract

This project aims to identify whether the introduction of routine preoperative risk assessment (RPRA) with the ‘National Emergency Laparotomy Audit (NELA) Calculator’ as standard‐of‐care for emergency major abdominal surgery at a regional Victorian hospital has improved postoperative outcomes and critical care unit (CCU) utilization. An audit was performed including all adult general surgery patients who required EMAS at Bendigo Health between September 2017 and August 2022, comparing outcomes before and after introduction of RPRA. RPRA reduced rate of unplanned CCU admissions but did not impact postoperative mortality or complication rates.

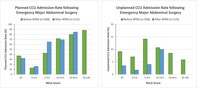

## Introduction

Emergency major abdominal surgery (EMAS), via open or minimally invasive approach, is associated with high mortality and morbidity.^1^, ^2^ The ANZELA‐QI bi‐national audit reports an average mortality rate of 6.2% following EMAS, which has been consistently declining since the commencement of the project.^3^, ^4^ EMAS audits from rural and regional Australia have demonstrated mortality ranging from 0% to 9.7%,^5^, ^6^, ^7^ comparable to the inter‐hospital mortality ranges reported by ANZELA (0–13.3%).^4^ Higher rurality hospitals typically have the lowest mortality, possibly due to lower acuity case‐mixes, higher degree of consultant surgeon involvement and ability to transfer to tertiary centres.^8^

The “National Emergency Laparotomy Audit (NELA) calculator” is a risk prediction tool developed using data from over 160 000 UK emergency laparotomy cases, which estimates perioperative mortality for EMAS as a percentage using preoperative patient characteristics.^1^ NELA has been shown to be a sensitive mortality risk prediction tool in Australian patients.^9^ Multiple Australian studies have demonstrated reduced mortality when routine preoperative risk assessment (RPRA) with objective risk prediction tools such as NELA are introduced as standard‐of‐care for EMAS.^4^, ^10^

RPRA allows preoperative identification of high‐risk patients and implementation of risk mitigation strategies, such as planned postoperative critical care unit (CCU) admission. Planned CCU admission facilitates early recognition and treatment of postoperative complications in the high‐risk EMAS patient, preventing deterioration and requirement for later unplanned CCU admission from the ward.^4^, ^11^ Therefore, both the Royal College of Surgeons and ANZELA‐QI recommend planned postoperative CCU admission for high risk EMAS patients.^4^, ^12^ This has been shown to improve postoperative mortality,^13^ and may also reduce CCU length of stay and costs.^10^ Despite these recommendations, there is high variation in CCU utilization for EMAS patients in Australian hospitals.^4^, ^8^

Avoidance of ‘futile’ procedures, where there is a low likelihood of meaningful postoperative recovery, is an important factor in reducing postoperative mortality for EMAS.^4^, ^14^ Objective scoring systems may aid the surgeon, family and patient in shared‐decision‐making regarding the appropriateness of proceeding to surgery as opposed to up‐front preoperative palliation. However no scores have been validated in this context, and there are no studies assessing NELA's utility in predicting ‘futile’ surgery.^15^

Bendigo Health is a 724‐bed regional hospital in northwest Victoria. RPRA with the NELA calculator was introduced as standard‐of‐care for EMAS patients at Bendigo Health in 2021. This project aims to identify whether the introduction of RPRA as standard‐of‐care has:

1. Improved perioperative mortality and morbidity
2. Impacted CCU admission rates
3. Impacted the proportion of patients palliated up‐front

## Methods

An audit was conducted including all patients assessed for EMAS by the General Surgery department at Bendigo Health between 1 September 2017 and 31 August 2022. We compared outcomes before and after the implementation of RPRA with the NELA risk calculator on 1 September 2021.

### Inclusion/exclusion criteria

Surgical patients were identified using the inclusion/exclusion criteria reported in the second ANZELA‐QI program summary report.^4^ Patients undergoing EMAS via open or minimally invasive approach were included. Laparoscopy or laparotomies performed for uncomplicated appendicitis, uncomplicated inguinal hernia, cholecystitis, trauma, or purely diagnostic purposes were excluded. Re‐operation within the same admission was considered a complication of the index procedure. Patients with surgical pathology requiring operative management who were assessed for EMAS but instead palliated up‐front due to high perioperative risk were also included. These are referred to as ‘*No‐lap’* patients.

### Case identification

Following the implementation of RPRA in September 2021, patients undergoing EMAS were prospectively identified by the treating team and details, including NELA score, documented in the digital medical record (DMR) as standard‐of‐care. Patients who underwent EMAS prior to implementation of RPRA were identified retrospectively by reviewing DMR of EMAS against inclusion/exclusion criteria. ‘No‐Lap’ patients were retrospectively identified by manually screening patients with high mortality diagnoses who were admitted under the general surgery team and did *not* undergo a subsequent operation. Patients who were palliated preoperatively due to unacceptably high perioperative risk, as decided by the patient, medical decision‐maker or clinician, were included in analysis.

### Outcomes

Variables were manually extracted from the digital medical record. Baseline characteristics included demographics and preoperative NELA score. For patients who underwent surgery prior to implementation of RPRA, or for whom NELA was not calculated and/or documented preoperatively, NELA was calculated retrospectively based on preoperative data as documented in the DMR. Primary outcomes were planned/unplanned CCU admission rates and 30‐day in‐hospital mortality. Secondary outcomes were severe postoperative complication rate and ‘No‐Lap’ rate. The endpoint employed was acute hospital discharge.

Postoperative complications were classified using the Clavien‐Dindo classification, and a grade of IIIa or more was considered a ‘severe’ complication.^16^ CCU admissions were classified as planned or unplanned based on the index admission. Admission to CCU directly from the theatre following the operation was considered planned. Where a patient was initially admitted to the ward, and then later admitted to CCU following deterioration or an unplanned return to theatre, the admission was considered unplanned.

### Analysis

A *P*‐value of less than 0.05 was considered statistically significant. Statistical testing was used to compare outcomes before and after introduction of RPRA. Chi‐squared test (2‐sided), Two Sample *t*‐test, and Mann–Whitney *U*‐test were used to analyze categorical, symmetrical continuous, and skewed continuous data respectively. Unadjusted logistic regression was used to determine whether NELA score was a significant predictor of patient outcomes. Subgroup analysis was performed for operative and Palliative subgroups. Analysis was performed using Posit ‘RStudio’ version 2023.12.1.

### Ethics

The Project was reviewed by the Bendigo Health Research Office and received Ethics Approval on 17/05/2023 (Reference QA/95640/BH‐2023‐368 286(v2)).

## Results

### Demographics

Six hundred and seventy‐three ‘operative’ patients who underwent EMAS, and 18 *‘No‐Lap’* patients were identified as meeting the inclusion criteria. 571 patients were identified in the 4 years prior to, and 120 in the year following the introduction of RPRA as standard‐of‐care.

Demographics are shown in Table 1. For the 691 included patients, the mean age was 65.20 (SD 16.55), and 371 (53.69%) patients were female. The median preoperative NELA score was 5 (IQR 1.5–11.75). There was no statistically significant difference before and after introduction of RPRA in Age (*P* = 0.71, 95% CI [−2.64, 3.88]), Sex (*P* = 0.36), ASA (*P* = 0.19), Diagnosis type (*P* = 0.20) or mean preoperative NELA score (*P* = 0.67, 95% CI [−1.93, 3.35]). There was also no difference in the distribution of NELA scores (*P* = 0.52).

**Table 1:** Patient demographics and preoperative characteristics

	All	Before RPRA†	Post RPRA	*P*‐value
	*n* = 691	*n* = 571	*n* = 120	
Age (Mean, SD)	65.20 (16.53)	65.30 (16.14)	64.68 (18.36)	0.7092‡
Sex (*n*, [%])				
Male	320 (46.31)	269 (47.11)	51 (42.50)	0.3572§
Female	371 (53.69)	302 (52.89)	69 (57.50)	
ASA (*n*, [%])				0.1896§
1E	29 (4.20)	27 (4.73)	2 (1.67)	
2E	106 (15.34)	90 (15.76)	16 (13.33)	
3E	345 (49.93)	287 (50.26)	58 (48.33)	
4E	198 (28.65)	155 (27.15)	43 (35.83)	
5E	13 (1.88)	12 (2.10)	1 (0.83)	
NELA Score				
Mean (SD)	9.80 (13.36)	9.92 (13.71)	9.21 (11.58)	0.6679¶
Median (IQR)	5 (1.5–11.75)	4.95 (1.6–11.53)	5.45 (1.1–12.4)	
NELA Range (*n*, [%])				
0–4.9	342 (49.49)	285 (49.91)	57 (47.50)	0.5229§
5–9.9	140 (20.26)	115 (20.14)	25 (20.83)	
10–19.9	109 (15.77)	87 (15.24)	22 (18.33)	
20–49.9	79 (11.43)	64 (11.21)	15 (12.5)	
50–100	21 (3.04)	20 (3.50)	1 (0.83)	

†Routine Preoperative Risk Assessment – Introduced on first September 2021. ‡Calculated with Independent t‐test. §Calculated with chi‐squared test. ¶Calculated with Mann Whitney *U*‐test.

### CCU utilization

**Fig. 1:**
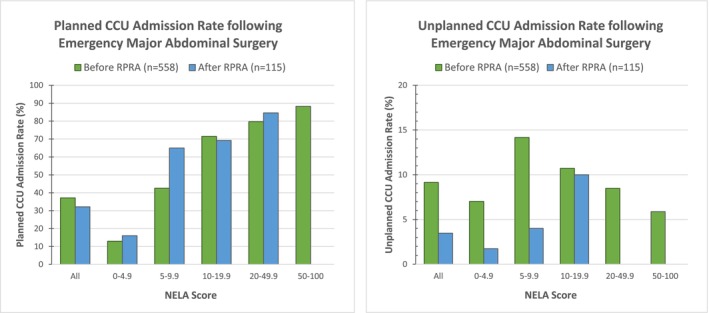
Planned and Unplanned CCU admission rates following Emergency Major Abdominal Surgery according to NELA score.

Thirty‐six percent (244/673) of the operative cohort had a planned postoperative CCU admission, with no significant difference following introduction of RPRA (37% before and 32% after, *P* = 0.3173). Higher NELA score was predictive of higher likelihood of planned postoperative CCU admission (OR = 1.14 [95% CI 1.111–1.171] *P* < 0.001), as shown in Figure 1.

There was a statistically significant reduction in the number of unplanned CCU admissions, from 9% (51/558) to 3.5% (4/115), following the introduction of RPRA (*P* = 0.04). Mean CCU length of stay was 1.88 days, with no significant difference between groups.

### Mortality

Amongst the 673 operative patients, 30‐day in‐hospital mortality was 5.20% (35/673). NELA score was a significant predictor of mortality. The likelihood of mortality increased by, on average, 6.6% for every 1% increase in NELA (OR = 1.066 [95% CI 1.048, 1.086] *P* < 0.001). There was no statistically significant difference in 30‐day Mortality following the introduction of RPRA (4.84% vs. 6.96%, *P* = 0.35).

Among all 691 patients *assessed* for surgery, 30‐day in‐hospital mortality was 7.09% (49/691). There was a statistically significant rise (*P* < 0.01) in 30‐day mortality from 6.30% (36/571) before RPRA to 10.83% (13/120) after RPRA, as shown in Table 2. NELA score was again a significant predictor of mortality (OR = 1.07, 95% CI 1.05–1.09), as illustrated in Figure S1.

**Table 2:** Comparison of outcomes amongst all patients assessed for emergency abdominal surgery before and after introduction of Routine Preoperative Risk Assessment

	All	Before RPRA†	After RPRA	*P*‐value
	*691*	*571*	*120*	
30 Day in‐hospital mortality (*n*, %)	49 **(7.09)**	36 **(6.30)**	13 **(10.83)**	*P* **= 0.000024**‡
	*n = 691*	*n = 571*	*n = 120*	
Preoperative palliation (*n*, %)	18 (**2.60**)	13 (**2.28**)	5 (**4.10**)	*P* **= 0.2537**‡
	*n = 18*	*n = 13*	*n = 5*	
30‐Day in‐hospital mortality following preoperative palliation (*n*, %)	14 (**77.77**)	9 (**69.23**)	5 (**100**)	*P* **= 0.1596**‡

†Routine preoperative risk assessment. ‡Calculated with chi‐squared test.

### Postoperative complications

As shown in Table 3, 32% (218/673) of operative patients experienced a severe postoperative complication and 8.02% (54/673) had an unplanned return to theatre. Mean hospital LOS following operation was 10.2 days. Likelihood of severe postoperative complication increased by, on average, 5.7% for every 1% increase in NELA (OR = 1.057 [95% CI 1.083–1.131] *P* < 0.01). There was no significant change in rates of severe complications, unplanned return‐to‐theatre and mean hospital LOS following introduction of RPRA (*P* = 0.20, *P* = 0.08, and *P* = 0.79, respectively).

**Table 3:** Comparison of postoperative outcomes following emergency major abdominal surgery before and after introduction of Routine Preoperative Risk Assessment

	NELA Score	All	Before RPRA†	Post RPRA	*P*‐value
*Number*		673	558	115	
30 day in‐hospital mortality *n* (%)	**All**	**35 (5.20)**	**27 (4.84)**	**8 (6.96)**	**0.35171**††
	0–4.9	1 (0.29)	1 (0.35)	0 (0.00)	
	5–9.9	3 (2.17)	2 (1.77)	1 (4.00)	
	10–19.9	9 (8.65)	6 (7.14)	3 (15.00)	
	20–49.9	18 (25.00)	14 (23.73)	4 (30.77)	
	50–100	4 (23.53)	4 (23.53)		
Severe‡ post‐operative complication *n* (%)	**All**	**218** (**32.39)**	**184** (**32.97**)	**34** (**29.57**)	**0.1967**††
	0–4.9	45 (13.16)	40 (14.04)	5 (8.77)	
	5–9.9	56 (33.33)	44 (38.94)	12 (48.00)	
	10–19.9	49 (40.58)	44 (52.38)	5 (25.00)	
	20–49.9	53 (50.96)	41 (69.49)	12 (92.31)	
	50–100	15 (88.24)	15 (88.24)		
Unplanned return to theatre *n* (%)	**All**	**54** (**8.02**)	**44** (**7.89**)	**10** (**8.70**)	**0.0848**††
	0–4.9	12 (3.51)	10 (3.51)	2 (3.51)	
	5–9.9	13 (9.42)	10 (8.85)	3 (12.00)	
	10–19.9	14 (13.46)	12 (14.29)	2 (10.00)	
	20–49.9	11 (15.28)	8 (13.56)	3 (23.08)	
	50–100	4 (23.53)	4 (23.53)		
Planned§ CCU admission *n* (%)	**All**	**244 (36.26)**	**207 (37.10)**	**37 (32.17)**	**0.3173**††
	0–4.9	41 (11.99)	37 (12.98)	4 (16.00)	
	5–9.9	61 (44.20)	48 (42.48)	13 (65.00)	
	10–19.9	69 (66.35)	60 (71.43)	9 (69.23)	
	20–49.9	58 (80.06)	47 (79.66)	11 (84.62)	
	50–100	15 (88.24)	15 (88.24)		
Unplanned¶ CCU admission *n* (%)	**All**	**55 (8.17)**	**51 (9.14)**	**4 (3.48)**	**0.0436**††
	0–4.9	21 (6.14)	20 (7.02)	1 (1.75)	
	5–9.9	17 (12.32)	16 (14.16)	1 (4.00)	
	10–19.9	11 (10.58)	9 (10.71)	2 (10.00)	
	20–49.9	5 (6.94)	5 (8.47)	0 (0)	
	50–100	1 (5.88)	1 (5.88)		
CCU LOS (days) Mean (SD)	**All**	**1.88 (3.97)**	**1.95 (4.18)**	**1.56 (2.78)**	**0.2123**‡‡
	0–4.9	0.48 (1.27)	0.53 (1.33)	0.23 (0.82)	
	5–9.9	2.42 (4.56)	2.42 (4.87)	2.40 (2.89)	
	10–19.9	2.94 (2.97)	3.27 (3.02)	1.60 (2.37)	
	20–49.9	5.11 (7.42)	4.98 (7.99)	5.69 (4.03)	
	50–100	5.59 (6.00)	5.59 (6.00)		
Hospital length of stay after surgery (days) Mean (SD)	**All**	**10.20** (10.95)	**10.17** (11.68)	**10.37** (6.38)	**0.7876**‡‡
	0–4.9	7.61 (6.70)	7.44 (6.93)	8.49 (5.34)	
	5–9.9	12.27 (13.14)	12.03 (14.22)	13.36 (6.49)	
	10–19.9	13.07 (13.16)	14.00 (14.14)	9.15 (6.78)	
	20–49.9	14.08 (15.33)	13.93 (16.73)	14.77 (6.22)	
	50–100	11.59 (11.97)	11.59 (11.97)		

†Routine preoperative risk assessment. ‡Clavien Dindo classification ≥ 3. §Direct CCU admission from theatre following index operation. ¶Indirect Postoperative CCU admission from ward or following unplanned return to theatre. ††Calculated with chi‐squared test. ‡‡Calculated with two sample (Welch's) *t*‐test.

### No‐lap patients

Eighteen of the 691 audited patients (2.60%) were palliated up‐front (Table 2). The *‘No‐lap’* rate rose from 2.28% (13/573) to 4.10% (5/120), which was not statistically significant (*P* = 0.25). Higher NELA score was predictive of higher likelihood of up‐front palliation (OR = 1.054 [95% CI 1.033–1.175] *P* < 0.001). 30‐day mortality amongst *‘no‐lap’* patients was 69% (9/13) before and 100% (5/5) after the introduction of routine preoperative risk assessment.

## Discussion

### CCU utilization

Unplanned CCU admission rate was significantly reduced following implementation of RPRA, which is consistent with previous studies.^4^, ^5^, ^11^ The reduction in unplanned admissions was not associated with an increase in planned CCU admissions, CCU LOS, or hospital LOS, suggesting RPRA aids in judicious use of critical care resources.

RPRA may reduce unplanned CCU admissions by prompting the following effects on patient care:

1. *Planned CCU admission for high‐risk patients*. Pre‐emptive admission of patients who will go on to have a CCU requirement avoids later unplanned admission. NELA and ANZELA‐QI both demonstrated a decline in unplanned CCU admission rate and an increase in planned CCU admissions following the introduction of RPRA.^4^, ^11^ In contrast this audit did not demonstrate any significant change in *planned* CCU admission rate. This may be due to high baseline CCU utilization, as prior to RPRA 76.25% of patients with NELA scores of ≥10 had planned CCU admissions, higher than the 64.6% reported by ANZELA‐QI.^4^
2. *Closer monitoring of high‐risk patients*. By prompting close monitoring of high‐risk patients, RPRA may facilitate earlier recognition and treatment of complications. This can prevent clinical deterioration to a level that requires an unplanned CCU admission, or “failure to rescue” the complication. Compared to planned CCU admissions, there was a trend toward longer hospital LOS amongst unplanned CCU admissions, (12 vs. 17 days, respectively, *P* = 0.08, 95% CI [0.7259, 9.0581]) which may reflect the delay in identification and treatment of the complication that resulted in the unplanned CCU admission.
3. *Earlier consideration of* ‘*ceiling of care*’. This may result in more high‐risk patients who are palliated up‐front, or who proceed to surgery but are deemed ‘not for CCU’, thus reducing later inappropriate, unplanned CCU admissions.

### Mortality and morbidity

The 5.20% post‐operative mortality rate was low when benchmarked against the 6.2% average mortality rate reported by ANZELA‐QI.^4^ Overall mortality, inclusive of “*no‐lap*” patients, was also comparable at 7.09%, despite the comparatively high mean NELA risk estimate of 9.80%. Mortality rate was also favourable when compared to regional Australian audits with comparable case‐mixes. Ho *et al*. reported a 9.7% 30‐day mortality rate following EMAS in a regional hospital with a mean predicted mortality risk estimate of 8.2%.^5^ More remote and rural hospitals typically report lower mortality, ranging from 0% to 4%, possibly due to lower acuity case mixes and a tendency to transfer high‐risk patients to tertiary centres.^6^, ^7^, ^17^ The severe complication rate of 32% was also favourable when benchmarked against the 40% reported by ANZELA‐QI.^4^

This audit validates the NELA calculator as an accurate mortality and morbidity risk prediction tool for a regional patient cohort, supporting the ongoing use of RPRA with NELA as standard‐of‐care. However, following implementation of RPRA, there was no significant change in postoperative 30‐day mortality, severe complication rate, or hospital LOS. This suggests RPRA predicts but does not improve postoperative outcomes. Therefore, RPRA is best utilized as part of a broader perioperative EMAS protocol.

There was a rise in overall mortality when including ‘*no‐lap’* patients. It is our opinion that this rise in mortality is unlikely to be a result of RPRA. This rise may instead reflect the growth of the health service and reduced need to transfer high acuity patients to tertiary centres, resulting in a higher observed mortality. Tertiary transfer rate was beyond the scope of this audit but should be included in future study.

### No‐lap patients

A strength of this study was the inclusion of ‘*no‐lap*’ patients. The preoperative palliation rate of 2.90% was lower than other EMAS audits, which reported rates between 4.1% and 8.3%.^10^, ^18^, ^19^ RPRA did not significantly impact the number of patients palliated up front, although there was a trend toward an increased *‘no‐lap’* rate post‐RPRA. The small sample size (*n* = 18) and retrospective case identification introduces the possibility of missing data which could account for the lower ‘*no‐lap’* rate seen in the pre‐RPRA group. Therefore, larger power prospective auditing is required to fully assess the impact of RPRA on up‐front palliation rate.

Whilst RPRA did not impact the ‘*no‐*lap’ rate, NELA was a significant predictor of postoperative outcomes and hence may be useful in guiding shared‐decision‐making discussions regarding the appropriateness of proceeding to surgery. Whilst NELA should not be the sole deciding factor, it can be used to inform patients and their families of likely surgical outcomes, and therefore inform decisions surrounding goals‐of‐care.

### Strengths and weaknesses

There were several limitations to this study, including retrospective identification of patients in the pre‐RPRA and palliative groups and a limited sample size in the post‐RPRA group. Furthermore, 90‐day mortality, discharge destination, and tertiary transfer rate are important outcomes that were beyond the scope of this audit. Finally, as a single‐centre regional study investigating hospital‐specific protocols, the external applicability of the results is limited, especially to metropolitan or more rural/remote hospitals.

## Conclusion

This study demonstrated regional hospitals can deliver favourable patient outcomes following EMAS. The study also demonstrates RPRA can improve CCU utilization for EMAS, without impacting postoperative mortality or morbidity. To further validate this, we plan to perform a cost‐effectiveness analysis of planned CCU admissions for high‐risk EMAS patients.

## Author contributions

**Jason Douglas Cox:** Data curation; investigation; project administration; writing – original draft; writing – review and editing. **Frank Dunley:** Data curation. **Jia Tian:** Formal analysis; software. **Kate Booth:** Project administration; writing – review and editing. **Jessica Paynter:** Writing – review and editing. **Chun Hin Angus Lee:** Conceptualization; methodology; supervision; writing – review and editing.

## Conflicts of interest

None declared.

## Supporting information


**Figure S1.** 30‐day Mortality Rate for all Patients Assessed for Emergency Major Abdominal Surgery according to NELA score.
