# Supplementary material for: Impact of routine pre‐operative risk assessment on patients undergoing emergency major abdominal surgery in a regional Victorian hospital
Source: ANZ J Surg. 2024 Oct 28;94(12):2238–44. doi: 10.1111/ans.19260 (PMC11713194; doi:10.1111/ans.19260)
Supplement: Supplementary file 1 — Figure S1. 30‐day Mortality Rate for all Patients Assessed for Emergency Major Abdominal Surgery according to NELA score. [file ANS-94-2238-s001.pdf]

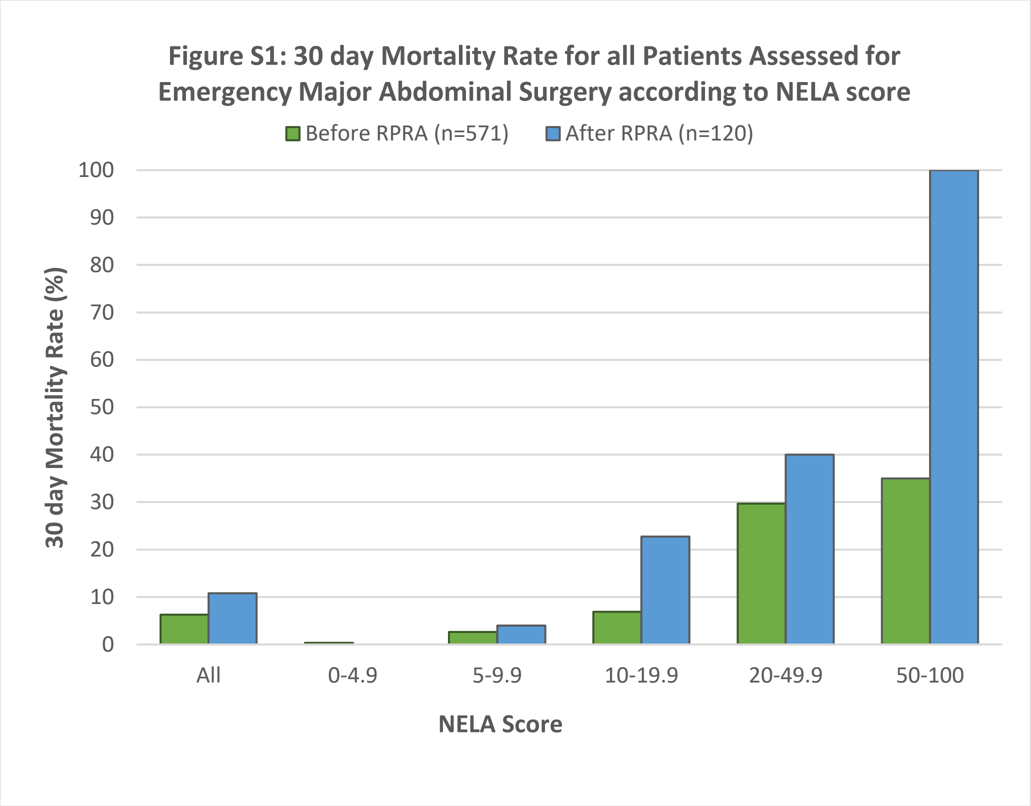
Figure S1: 30 day Mortality Rate for all Patients Assessed for Emergency Major Abdominal Surgery according to NELA score
Before RPRA (n=571)
After RPRA (n=120)
30 day Mortality Rate (%)
100
90
80
70
60
50
40
30
20
10
0
All
0-4.9
5-9.9
10-19.9
20-49.9
50-100
NELA Score
